# Obesity and cellulitis: Associations with infection severity, recurrence, and healthcare utilization—A systematic review

**DOI:** 10.1016/j.obpill.2026.100345

**Published:** 2026-10-13

**Authors:** Fariha Hanif, Toni-Ann Lewis, Michael Kaiser

**Affiliations:** a Department of Internal Medicine, SUNY Downstate Health Sciences University, 450 Clarkson Avenue, Brooklyn, NY, 11203, USA; b NewYork-Presbyterian Brooklyn Methodist Hospital, 506 6th Street, Brooklyn, NY, 11215, USA

**Keywords:** Body mass index, Cellulitis, Healthcare utilization, Obesity, Skin and soft tissue infection, Systematic review

## Abstract

**Background:**

Obesity is a growing global health concern associated with chronic inflammation, immune dysregulation, and impaired skin barrier function, all of which may increase susceptibility to cellulitis and influence clinical outcomes. This systematic literature review synthesized current evidence examining the relationship between obesity and cellulitis risk, infection severity, and healthcare utilization.

**Methods:**

This PRISMA 2020-guided systematic review and narrative synthesis searched PubMed/MEDLINE, Embase, CINAHL, and Web of Science for English-language primary studies published from January 2015 through December 2025. Eligible studies involved adults with cellulitis or erysipelas—or adult population cohorts reporting a separable cellulitis/erysipelas-specific outcome—evaluating body mass index (BMI) or obesity as an exposure or predictor. Target outcomes comprised cellulitis development or hospitalization, recurrence, treatment response or failure, severity, length of stay, readmission, cost, and mortality. Given the substantial heterogeneity across study designs and outcome definitions, findings were synthesized narratively. When sufficiently complete primary reports were available, risk of bias was evaluated using design-appropriate Joanna Briggs Institute (JBI) critical appraisal tools (Appendix 4).

**Results:**

Eighteen primary studies met the eligibility criteria. Across population cohorts and cellulitis-specific clinical studies, higher BMI or obesity was generally associated with greater cellulitis risk (e.g., ORs ranging from 1.11 to 4.7) and, in several studies, recurrence or increased healthcare utilization (such as prolonged length of stay). However, associations were not uniform: some studies reported null findings for treatment failure or readmission. The evidence base was predominantly observational and methodologically heterogeneous, so conclusions regarding downstream clinical outcomes are less certain than those regarding cellulitis risk and incidence.

**Conclusion:**

Obesity is an important risk marker of cellulitis susceptibility and adverse clinical outcomes. Future studies using standardized outcome measures and improved assessments of adiposity beyond BMI are needed to guide prevention strategies and optimize management of this growing population.

## Graphical abstract

**Figure:**
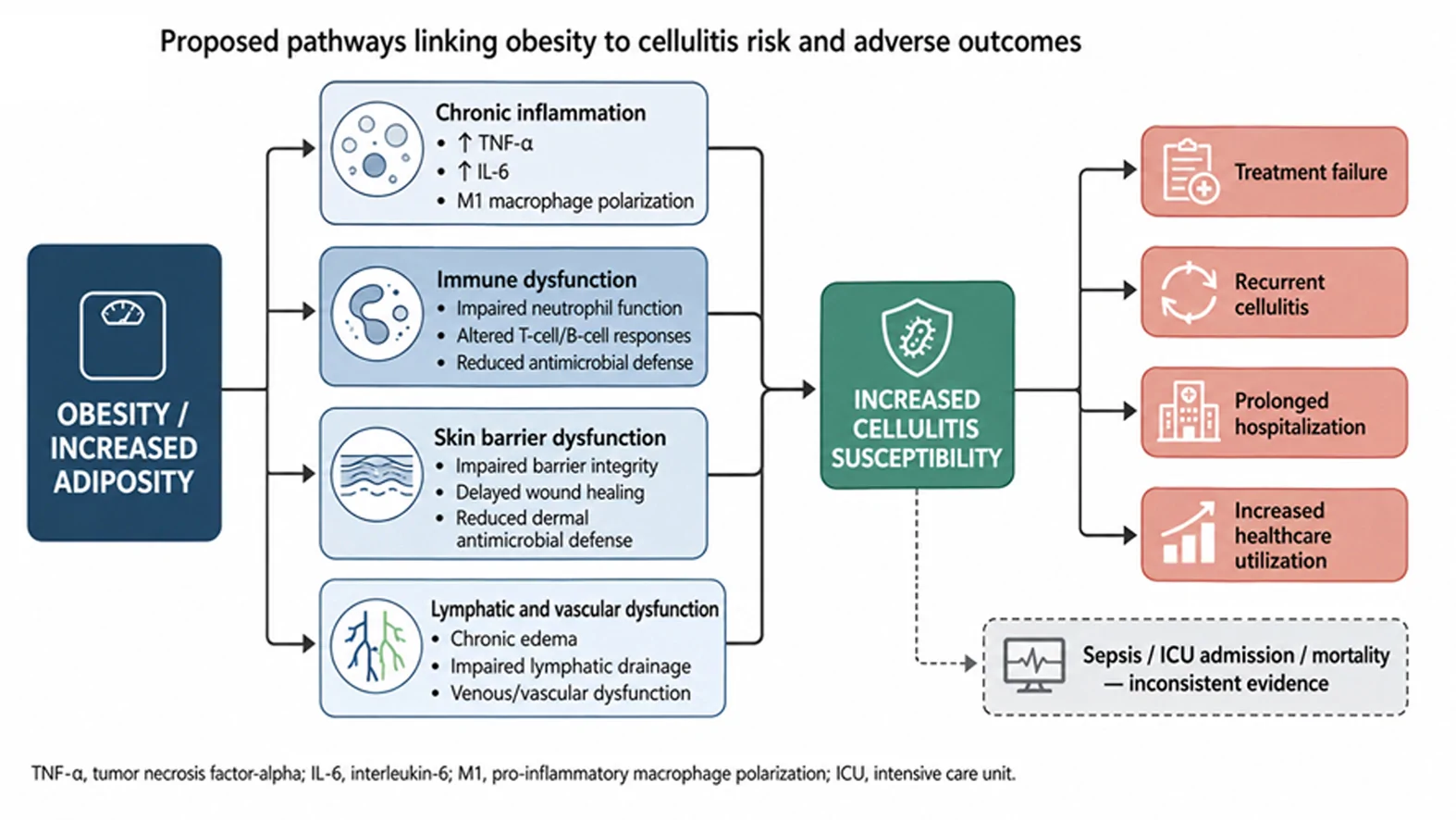

## 1 Introduction

### 1.1 Epidemiology of cellulitis

Cellulitis is an infection of the deep dermis and subcutaneous tissue that is a significant global health burden, with more than 650,000 admissions per year in the United States and estimated 14.5 million cases annually accounting for $3.7 billion in ambulatory care costs [1]. The number of hospitalizations for cellulitis and abscesses in the United States has also been on the rise, increasing by 73% from 1997 to 2011, and the readmission rate following a hospitalization for cellulitis within 30 days is approximately 9.8% [1], [2]. These statistics highlight the substantial clinical and economic impact of cellulitis.

### 1.2 Obesity and infection risk

The prevalence of obesity continues to rise globally, with obesity now being recognized as an independent risk factor for the development and severity of a wide range of infectious diseases [3]. Skin and soft tissue infections are among the predominant infection types reported in this population [3]. A study using UK Biobank data supported a causal association between higher body mass index and increased risk of hospital admission for infectious disease, including skin and soft tissue infection (OR 1.11 per standard deviation increase in BMI; 95% CI 1.09–1.12) [4].

### 1.3 Adipose tissue-derived cytokines

Obesity represents a state of chronic, low-grade systemic inflammation, driven primarily by the endocrine and immunological activity of expanded adipose tissue [5], [6]. As adipocytes undergo hypertrophy in states of sustained caloric excess, they demonstrate altered secretory activity with increased release of pro-inflammatory cytokines [7]. Key pro-inflammatory cytokines released from adipose tissue are tumor necrosis factor-alpha (TNF-α), interleukin 6 (IL-6), monocyte chemoattractant protein-1 (MCP-1/CCL-2) and IL-1β [8]. In addition to production by hypertrophied adipocytes, infiltrating macrophages within adipose tissue also release these pro-inflammatory cytokines. In obesity, the macrophages in adipose tissue shift from an anti-inflammatory M2 polarization to a pro-inflammatory M1 polarization, further promoting systemic inflammation [9]. Body adipose tissue mass is estimated to be responsible for 15–35% of systemic IL-6, and both IL-6 and TNF-α serum levels are elevated in patients with obesity and decrease with weight loss [10].

TNF-α plays the most crucial role in this inflammatory cascade, by promoting the secretion of IL-6, inducing adipocyte apoptosis, and activating the NF-kB signaling pathway, which further amplifies oxidative stress and cytokine release. Secretion of IL-6 drives the acute-phase response (C-reactive protein, fibrinogen, complement C3), contributing to systemic inflammation even in the absence of acute infection [8], [11]. This inflammatory state impairs neutrophil chemotaxis, reduces natural killer cell activity, dysregulates T-cell responses, and compromises B-cell function [12], [13]. When individuals with obesity experience an acute infection, they may mount an exaggerated cytokine storm but show delayed pathogen clearance.

Additionally, obesity results in loss of dermal adipocyte progenitors that produce antimicrobial peptides and poses pharmacokinetic challenges with standard antibiotic dosing, including altered volume of distribution and subtherapeutic tissue penetration under standard fixed antibiotic dosing [14], [15]. Despite this comprehensive understanding of obesity as a state of chronic inflammation, no comprehensive literature review has synthesized the evidence on the dose-response relationship between BMI and the full spectrum of cellulitis severity and utilization outcomes. These proposed pathways and their downstream clinical consequences are summarized in Fig. 2.

### 1.4 Objectives

The primary objective of this literature review is to synthesize and critically appraise current evidence on the association between BMI (obesity) and: (1) infection severity (sepsis and surgical intervention); (2) hospital utilization (length of stay, readmission, and cost); and (3) treatment outcomes (mortality and clinical nonresponse). Secondary objectives include exploring biological mechanisms linking adiposity to cellulitis outcomes and identifying gaps for future research.

## 2 Methods

This was a systematic literature review conducted according to the Preferred Reporting Items for Systematic Reviews and Meta-Analyses (PRISMA) 2020 framework. The completed PRISMA 2020 Checklist is provided as Appendix 1. The eligibility criteria were refined during manuscript preparation to ensure that included studies directly addressed the prespecified review question. The review protocol was not prospectively registered (e.g., in PROSPERO).

**Eligibility criteria:** English-language primary studies published from January 2015 through December 2025 were eligible if they included adults (age ≥ 18 years) with cellulitis or erysipelas, or an adult population cohort reporting a separable cellulitis/erysipelas-specific outcome. BMI or obesity had to be evaluated as an exposure or predictor in relation to at least one prespecified clinical outcome: cellulitis development/incidence, cellulitis-related hospitalization, recurrence, infection severity, treatment response or failure, length of stay, readmission, healthcare cost, or mortality. Observational cohort, case-control, cross-sectional, and interventional primary studies were eligible from inpatient, emergency, outpatient, and population-based settings.

**Exclusion criteria:** Reviews, editorials, case reports, and conference abstracts without a full peer-reviewed report were excluded from the primary evidence synthesis. Studies were excluded when they focused on broad skin and soft-tissue infection (SSTI) or general infection without a separable cellulitis/erysipelas-specific outcome; postoperative surgical-site infection; necrotizing soft-tissue infection; or mixed pediatric/adult populations without separable adult data. Studies restricted to patients with obesity or high body weight were excluded when obesity/BMI was not itself evaluated as the comparative exposure or predictor. Studies in which obesity was reported only descriptively, without an analyzed relationship to a prespecified cellulitis outcome, were not included in the outcome synthesis.

### 2.1 Search strategy

A comprehensive electronic search was performed in July 2026 across PubMed/MEDLINE, Embase, Web of Science, and CINAHL for peer-reviewed articles published from January 2015 through December 2025. The December 2025 search cutoff was established to ensure complete 12-month calendar year indexing stability across all four databases. Search strategies combined controlled vocabulary (Medical Subject Headings [MeSH] in PubMed and Emtree terms in Embase) with free-text terms addressing obesity/adiposity and cellulitis/erysipelas or relevant infection outcomes. Boolean operators (AND and OR) were used, and the strategy was adapted to each database while retaining the same core concepts. The database-specific search strategies are provided in Appendix 2.

Retrieved records were imported into Rayyan for duplicate removal and screening. Reference lists of included articles and key review papers were manually searched. Study selection steps are summarized in the PRISMA flow diagram (Fig. 1).

**Fig. 1:**
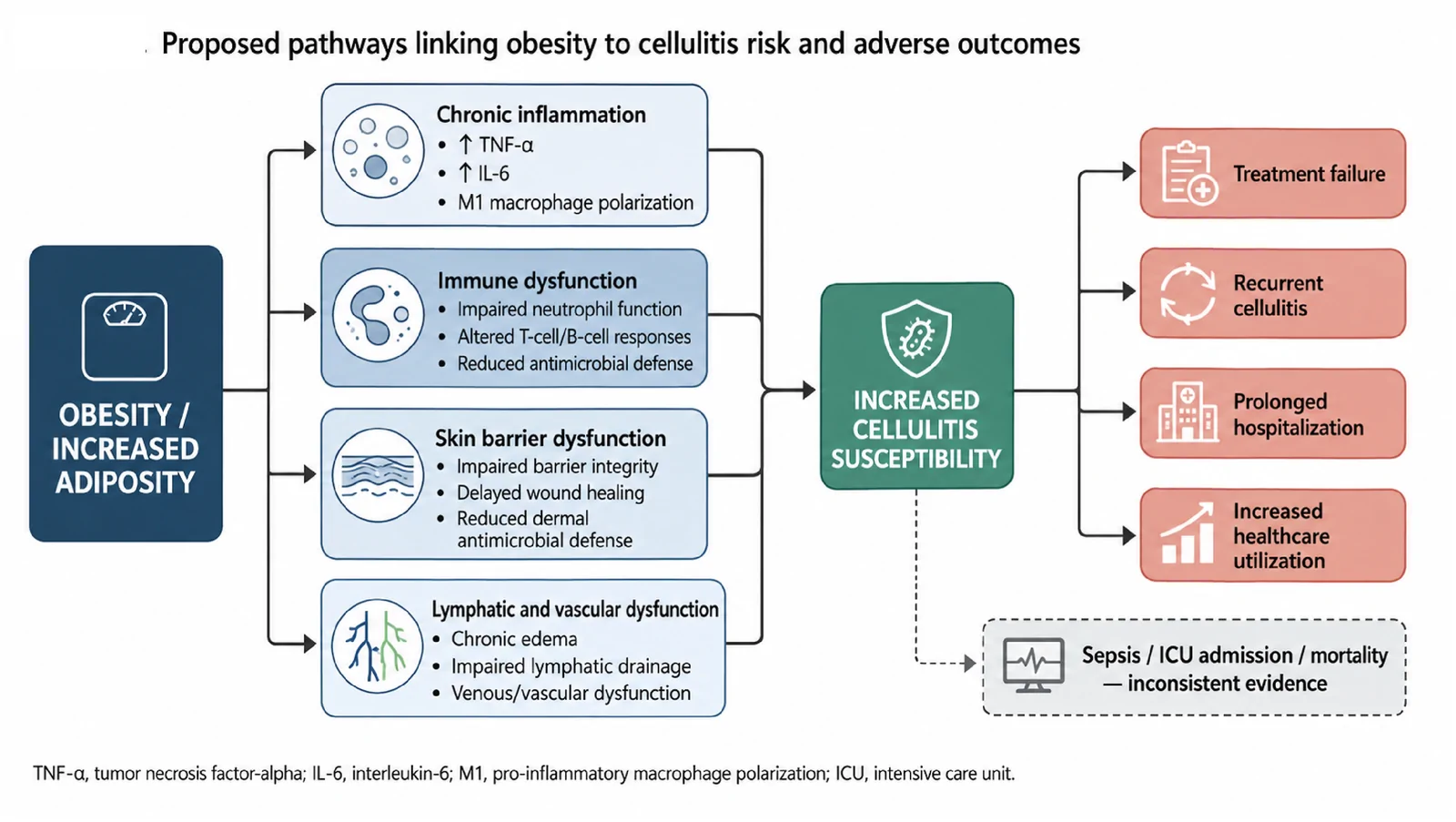
Preferred Reporting Items for Systematic Reviews and Meta-Analyses (PRISMA) flow diagram of study selection.

**Fig. 2:**
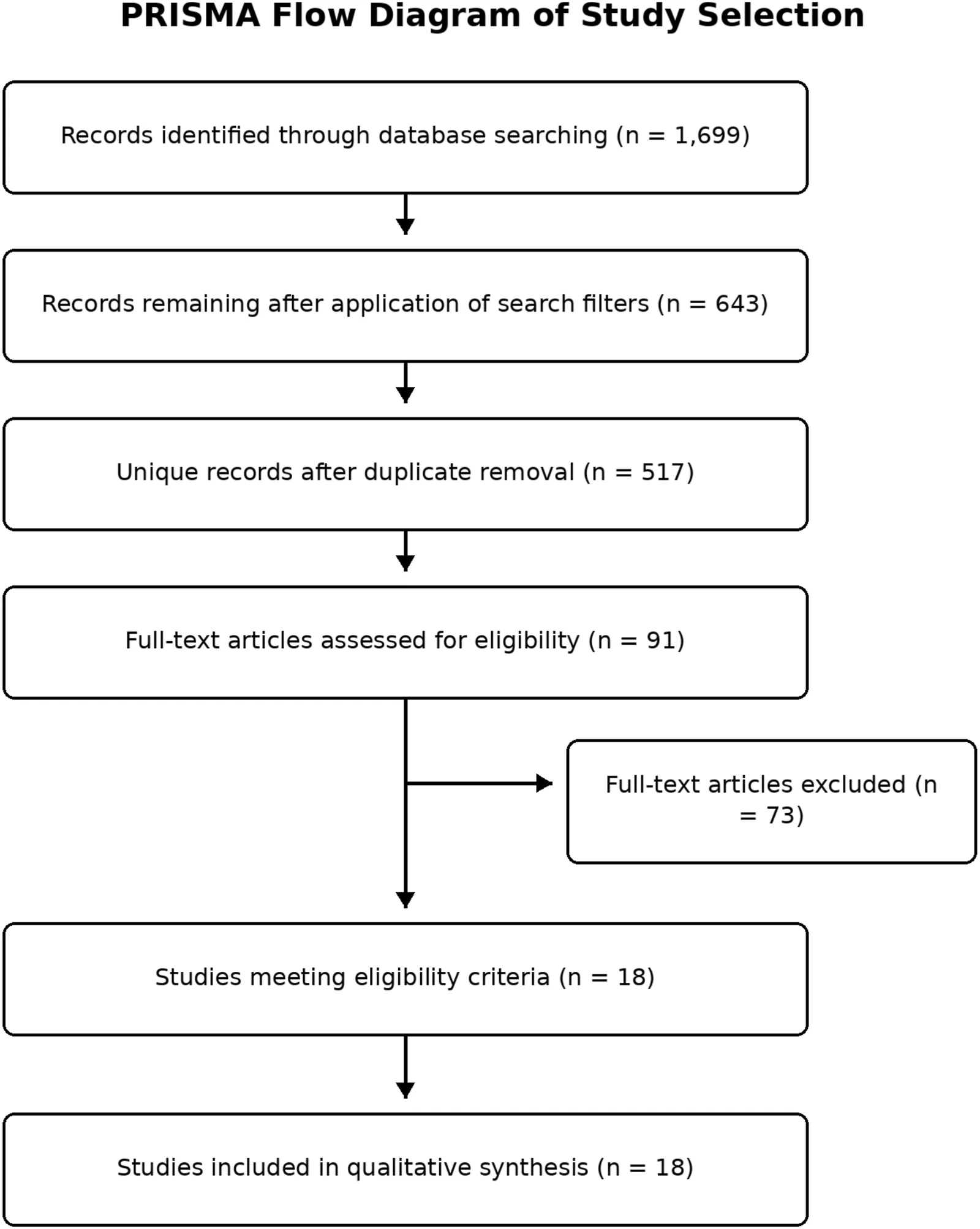
Proposed pathways linking obesity to cellulitis risk and adverse outcomes. TNF-α, tumor necrosis factor-alpha; IL-6, interleukin-6; ICU, intensive care unit.

### 2.2 Data extraction

Data were extracted into a structured evidence table. Extracted variables included study characteristics (author, publication year, country, study design, sample size, population and care setting), participant characteristics (age, sex, race/ethnicity, BMI distribution, comorbidities), exposure definitions (BMI/obesity categories, cellulitis/erysipelas definitions), infection severity (sepsis, surgical interventions), and biological mechanisms or study limitations where available. Outcomes were grouped into cellulitis development, disease severity, treatment response/failure, healthcare utilization (length of stay, readmission, cost, and mortality). Null findings were retained alongside statistically significant associations.

Primary study selection and data extraction were conducted by one reviewer and independently audited and validated by secondary reviewers. Feasibility of meta-analysis was formally evaluated; due to extreme clinical and methodological heterogeneity in BMI categorizations, outcome definitions, and statistical controls, quantitative pooling was deemed inappropriate, and a narrative synthesis was conducted.

### 2.3 Risk-of-bias assessment

Risk of bias was assessed using design-appropriate Joanna Briggs Institute (JBI) critical appraisal instruments: the JBI Checklist for Cohort Studies for cohort designs, the JBI Checklist for Case Control Studies for case-control studies, and the JBI Checklist for Analytical Cross Sectional Studies for cross-sectional designs (Appendix 4). Appraisal focused on participant selection and comparability, validity and reliability of exposure and outcome measurement, identification and management of confounding, completeness of follow-up where applicable, and appropriateness of statistical analysis. Judgments were based on the published primary report; when only an abstract or otherwise incomplete report was available, the appraisal was classified as provisional or pending rather than assigning favorable ratings to unreported domains. Of the 18 retained studies, 12 had sufficiently detailed primary reports for item-level appraisal, five were assigned provisional assessments because only partially accessible reports were available, and one remained pending because the accessible report did not provide enough methodological detail for a defensible item-level assessment. Unreported methodological domains were classified as unclear rather than assumed to be low risk.

### 2.4 Registration, protocol, and review-process amendments

The review was not prospectively registered, and a formal protocol was not prepared before screening. The eligibility criteria were refined during manuscript preparation to ensure alignment between the review question and the studies included in the final synthesis. This post hoc refinement is acknowledged as a methodological limitation.

## 3 Results

The database search yielded 1699 initial records (643 after automated database filters). Following duplicate removal, 517 unique records underwent title/abstract screening, yielding 91 full-text articles for eligibility evaluation. Ultimately, 18 primary studies met all criteria and were included. Findings were heterogeneous and included both positive and null associations. Database search counts are reported in Table 1, and characteristics of the 18 retained studies are summarized in Appendix 3.

**Table 1:** Summary of literature review findings.

Database	# of articles before filtering for year, language, age and human species	# of articles after filtering for year, language, age, and human species
PubMed/MEDLINE	153	73
Embase	1029	323
CINAHL	243	97
Web of Science	274	150

Note: The final review includes 18 studies meeting the eligibility criteria.

### 3.1 Study selection

Of 91 articles assessed at full text, 73 were excluded and 18 studies met the eligibility criteria and were included in the final qualitative synthesis. The study-selection process is summarized in Fig. 1.

### 3.2 Obesity and risk of cellulitis

Cheong et al. reported a graded association between increasing BMI and both incident cellulitis and cellulitis-related hospitalization in a large prospective cohort [16]. Nassaji et al. likewise found higher BMI associated with acute cellulitis in a case-control study [17].

Population-based evidence also supports the association. Harpsøe et al. reported increasing risk of erysipelas with higher BMI in a prospective cohort of Danish women [18], while Cheong et al. found increasing BMI associated with incident cellulitis and cellulitis-related hospitalization after multivariable adjustment [16].

In a hospital-based case-control study from Cameroon, Njim et al. found obesity associated with lower-limb cellulitis in univariable analysis (OR 3.0; 95% CI 1.5–6.0) and after multivariable adjustment (adjusted OR 4.7; 95% CI 1.5–14.7) [19].

Other retained studies illustrate both positive and null associations across different cellulitis outcomes. Wiederhold et al. reported an association between obesity and cellulitis diagnosis in a prospective emergency-department cohort [20]. Sapuła et al. associated higher BMI with recurrent erysipelas/cellulitis [21], whereas Chamli et al. did not identify BMI as a significant predictor of recurrence in their cohort [22]. In a large electronic-health-record cohort, Carey et al. found a strong positive association between increasing BMI and cellulitis incidence [23].

Collectively, the available literature supports higher BMI or obesity as an important risk factor for cellulitis development, although effect estimates and populations vary across studies [16], [17], [18], [19], [23].

### 3.3 Obesity and infection severity

In addition to increasing the risk of developing cellulitis, obesity may adversely affect selected clinical outcomes once cellulitis occurs. Across the retained studies, reported associations included treatment failure or early nonresponse, prolonged antimicrobial therapy, prolonged hospitalization, and short-stay overstay or transfer of care; however, findings were not uniform across outcomes.

Theofiles et al. reported that severe obesity was associated with greater outpatient cellulitis treatment failure, while Matych et al. identified obesity as an independent factor associated with prolonged hospitalization for erysipelas [24], [25].

Evidence for downstream severity and treatment outcomes was more heterogeneous. In an inpatient/observation cohort treated with cephalexin, Kaufman et al. found no statistically significant difference in therapeutic failure between patients with severe obesity and patients without (20.0% vs 14.5%; P = 0.53), although the study was underpowered [26]. Conversely, Bruun et al. associated higher BMI with poorer early treatment response [27], while Abetz et al. identified obesity as a predictor of short-stay overstay or transfer of care [28]. These mixed findings suggest that obesity alone may not consistently predict treatment response or clinical failure across cellulitis outcomes.

The retained studies used different definitions of severity and treatment failure, including early clinical nonresponse, need for additional or prolonged antimicrobial therapy, hospital transfer/overstay, and composite treatment-failure outcomes. This heterogeneity, together with the predominance of observational designs and lack of accounting for critical clinical confounders (such as antimicrobial resistance patterns, blood or tissue culture results, drug allergies, or non-weight-adjusted antibiotic dosing), limits direct comparison and precluded quantitative pooling of downstream clinical outcomes.

### 3.4 Obesity and hospital utilization

Several cellulitis-specific studies linked obesity with greater healthcare utilization. Kozłowska et al. reported that patients hospitalized for more than 10 days were more likely to have higher BMI [29], and Matych et al. identified obesity as an independent factor associated with prolonged hospitalization [25], and Grinlinton et al. likewise reported an association between obesity and longer hospital length of stay [30]. Abetz et al. found obesity independently associated with transfer of care or overstay after admission to an emergency short-stay unit (adjusted OR 4.33, 95% CI 1.38–15.59) [28]. Garg et al. likewise identified obesity as a factor associated with decreased likelihood of discharge among patients admitted with lower-limb cellulitis [31].

Not all utilization outcomes were consistently associated with obesity. In Garg et al.'s readmission analysis, obesity was not more frequent among patients readmitted for lower-limb cellulitis [32]. Zhang et al. reported obesity among factors associated with longer outpatient parenteral antimicrobial therapy duration [33], but the available report provides limited methodological detail and this study's risk-of-bias assessment remains pending. Taken together, the evidence is more consistent for prolonged treatment or length of stay than for readmission.

Overall, obesity appears to be associated with increased cellulitis-related healthcare utilization in several settings, but effect estimates and outcome definitions vary. The presence of both positive and null studies supports a cautious interpretation: obesity is a clinically relevant risk marker, but its independent association differs by outcome, population, and study design.

## 4 Discussion

### 4.1 Principal findings

This systematic literature review synthesizes evidence on obesity across the cellulitis clinical continuum, including disease development, recurrence, treatment response, and healthcare utilization. The most consistent evidence supports an association between increasing adiposity and cellulitis/erysipelas incidence and prevalence. Evidence for downstream outcomes after cellulitis develops is directionally concerning but more heterogeneous, with positive associations for recurrence, early nonresponse, prolonged therapy, and length of stay in some studies and null findings for selected treatment-failure and readmission outcomes.

Perhaps the most consistent finding across the reviewed literature was the association between obesity and an increased risk of developing cellulitis. Multiple large cohort and population-based studies demonstrated a dose-dependent relationship between increasing BMI and cellulitis incidence/hospitalizations, with obesity remaining statistically significant even after adjustment for important possible confounders like diabetes mellitus and metabolic health status. These findings suggest that obesity itself is an independent risk marker for cellulitis rather than a surrogate marker for other metabolic diseases. Although one study described a “U-shaped” relationship between BMI and infection-related hospitalizations, this investigation evaluated infections broadly rather than cellulitis specifically. In contrast, studies focused exclusively on cellulitis/skin and soft tissue infections consistently demonstrated a positive association between increasing BMI and cellulitis risk, strengthening the evidence for obesity as a clinically meaningful risk factor.

The observed epidemiologic associations are biologically plausible given the well-established pathophysiologic consequences of obesity. As discussed previously, obesity-induced chronic inflammation, immune dysregulation, impaired lymphatic function, and skin barrier dysfunction provide biologically plausible mechanisms linking obesity to increased cellulitis susceptibility.

Moreover, obesity is associated with impaired skin barrier integrity, diminished production of antimicrobial peptides by dermal adipocytes, delayed wound healing, chronic edema, and lymphatic dysfunction, all of which facilitate bacterial entry and impair infection resolution [14], [15], [34]. Collectively, these mechanisms provide a biologically coherent explanation for the increased incidence of cellulitis observed across multiple epidemiologic studies.

Once cellulitis develops, obesity may influence selected clinical outcomes, but the evidence is not uniform. Higher BMI or obesity was associated with recurrence in some erysipelas/cellulitis cohorts, poorer early response in a prospective hospitalized cohort, and greater utilization in several inpatient and short-stay studies. In contrast, a small underpowered cephalexin cohort did not identify a statistically significant increase in treatment failure among patients with morbid obesity, and a lower-limb cellulitis cohort did not find obesity to be more frequent among readmitted patients. These null findings are important and temper any conclusion that obesity universally worsens the clinical course of cellulitis.

Interpretation is further limited by heterogeneity in outcome definitions and study design. The retained literature includes prospective and retrospective cohorts, case-control studies, and cross-sectional analyses, with different BMI thresholds and different measures of recurrence, treatment failure, response, and utilization. The risk-of-bias appraisal therefore emphasizes confounding control, exposure/outcome measurement, participant selection, and completeness of follow-up rather than treating all studies as equally informative.

Healthcare utilization is a particularly relevant extension of the obesity-cellulitis relationship. Several studies associated obesity with prolonged hospitalization, reduced likelihood of discharge, short-stay overstay or transfer of care, or longer parenteral therapy. However, readmission findings were not consistently positive. The overall evidence therefore supports obesity as a potential contributor to resource use while underscoring the need for prospective studies using standardized utilization outcomes and more granular measures of adiposity.

### 4.2 Implications

The findings of this review have several important clinical implications. First, clinicians should recognize obesity as an independent risk factor for cellulitis development and recurrence rather than attributing increased infection risk primarily to associated comorbidities such as diabetes. Preventive interventions include weight reduction, meticulous skin care, early treatment of fungal infections, management of chronic edema and lymphedema, and patient education regarding skin barrier/integrity may reduce cellulitis incidence among high-risk individuals. Second, given the heterogenous evidence regarding treatment outcomes, statements regarding monitoring should be qualified cautiously: while evidence is strongest for cellulitis incidence and recurrence, patients with obesity presenting with cellulitis may benefit from closer clinical monitoring due to potential risks of early treatment nonresponse, prolonged therapy, or elevated healthcare resource utilization reported in selected cohorts.

### 4.3 Limitations

The evidence base is predominantly observational, and residual confounding is an important limitation. Although several large cohorts adjusted for metabolic and demographic covariates, smaller retrospective studies varied in their handling of confounding and were more susceptible to selection and measurement bias. Crucially, few primary studies controlled for key clinical confounders such as antimicrobial resistance patterns, blood or tissue culture results, drug allergies, or non-weight-adjusted antibiotic dosing, all of which significantly influence treatment response independent of BMI. The current JBI-based appraisal identifies the strongest support in large prospective or linked-data cohorts, while many smaller cellulitis-specific studies provide moderate-strength evidence. A small number of studies remain provisional or pending because sufficiently detailed primary reports were not accessible for complete item-level appraisal.

This review also has methodological limitations. The eligibility criteria were refined during manuscript preparation rather than through a prospectively registered protocol amendment. In addition, the original study-level screening records for 14 articles excluded during the initial full-text review were unavailable; consequently, the specific historical reasons for exclusion of these records could not be verified, presenting potential implications for reproducibility and selection bias.

The search strategy was executed in July 2026 but eligibility was restricted to literature published between January 2015 and December 2025. Because search terms focused heavily on hospitalization, severity, length of stay, and treatment failure, the search strategy may have missed studies examining cellulitis incidence, recurrence, or risk factors that did not explicitly index these utilization terms. Additionally, excluding January–July 2026 studies may have omitted recently published literature. Only English-language publications were considered, introducing potential language bias. Publication bias is also possible, particularly because statistically significant obesity associations may be more likely to be reported. Nevertheless, the synthesis deliberately retains null findings where available, including studies reporting no statistically significant association between morbid obesity and cephalexin treatment failure or between obesity and cellulitis readmission.

An additional limitation of the current evidence is the widespread reliance on BMI as the primary measure of obesity. BMI does not differentiate adipose tissue distribution or body composition and inadequately reflects visceral adiposity, waist circumference, metabolic health phenotypes (e.g., metabolically unhealthy normal weight vs. metabolically healthy obesity), or sarcopenic obesity, all of which exhibit stronger biological relationships with immune dysregulation, chronic inflammation, and infection risk than BMI alone. Future studies incorporating measures such as waist circumference, waist-to-hip ratio, or body composition analyses may provide a more accurate assessment of obesity-related cellulitis risk.

Despite these limitations, the review has several strengths. It uses a multi-database search compliant with PRISMA 2020 guidelines (Appendix 1), a clearly defined cellulitis/erysipelas-specific eligibility framework, and design-appropriate JBI critical appraisal. The review also extends beyond the already established association between obesity and cellulitis susceptibility by emphasizing recurrence, treatment response, and healthcare utilization, areas in which the evidence is clinically important but less consistently synthesized.

### 4.4 Future directions

Given these limitations, future research should prioritize well-designed prospective multicenter studies employing standardized definitions of obesity, cellulitis severity, treatment failure, and recurrence. Incorporation of more comprehensive measures of adiposity, including waist circumference, waist-to-hip ratio, and body composition analyses, may improve risk stratification beyond BMI alone. Formal quantitative meta-analyses or subgroup analyses should also be assessed for feasibility when sufficiently homogenous cohorts exist. Additional research is also needed to determine whether interventions targeting obesity, including structured weight-loss programs and pharmacologic therapies, reduce cellulitis incidence, recurrence, and healthcare utilization.

An additional area warranting future investigation due to its growing popularity is the potential role of glucagon-like peptide-1 receptor agonists (GLP-1RAs) in cellulitis management. Beyond promoting weight loss, current literature has shown that GLP-1RAs can improve glycemic control, reduce systemic inflammation, and favorably influence several obesity-related metabolic abnormalities [35]. Emerging evidence further suggests that these agents may also improve endothelial function and reduce lower extremity edema, all of which could theoretically lower susceptibility to cellulitis and recurrent skin/soft tissue infections [36], [37]. However, this section remains speculative, as direct evidence linking GLP-1 RAs to reduced cellulitis incidence, recurrence, or treatment failure remains limited. Future prospective studies should investigate whether pharmacologic weight reduction translates into lower cellulitis incidence, improved clinical outcomes, and reduced healthcare utilization in high-risk populations.

## 5 Conclusion

Obesity is consistently associated with increased susceptibility to cellulitis and erysipelas, with large population cohorts supporting a dose-related relationship between higher BMI and cellulitis-specific outcomes. Evidence also suggests that obesity may contribute to recurrence, treatment nonresponse, prolonged therapy, and greater healthcare utilization, although these downstream associations are less consistent and are derived largely from observational studies. Null findings for selected treatment-failure and readmission outcomes underscore the need for cautious interpretation. Future prospective multicenter studies should use standardized cellulitis definitions, consistent clinical outcomes, and measures of adiposity beyond BMI to clarify which obesity-related factors are most predictive of adverse cellulitis outcomes.

Several limitations within the existing evidence base highlight the need for higher-quality prospective research. Future studies should incorporate more comprehensive measures of adiposity beyond BMI, standardize clinically relevant outcome measures, and evaluate whether weight reduction and optimized management of obesity-related complications can reduce cellulitis incidence and recurrence.

As the global prevalence of obesity continues to rise, the burden of cellulitis is also likely to increase, with important implications for patients and healthcare systems alike. Recognizing obesity not merely as a comorbidity, but as an independent risk marker associated with cellulitis development and disease progression, may facilitate earlier identification of high-risk individuals, implementation of targeted preventive strategies, and more individualized clinical management. Ultimately, improving our understanding of the relationship between obesity and cellulitis has the potential to reduce recurrent infections, healthcare utilization, and the growing economic burden associated with this common infectious disease.

## 6 Key clinical takeaways

• Obesity should be recognized as an independent risk factor for cellulitis development and recurrence.
• Patients with obesity and cellulitis may warrant tailored management and monitoring for treatment failure, prolonged therapy, recurrence, and increased healthcare utilization, though routine assumption of treatment failure is unwarranted.
• Future studies should evaluate adiposity measures beyond BMI and determine whether intentional weight reduction reduces cellulitis incidence and recurrence.

## Author contributions

Fariha Hanif, MPH: literature search, screening, data extraction, manuscript drafting and revision. Toni-Ann Lewis, MD, MPH: mentorship, conceptualization, supervision, critical review, and manuscript revision. Michael Kaiser, MD, MPH: conceptualization, supervision, validation, and manuscript review and editing. All authors reviewed and approved the final manuscript.

## Ethical adherence and ethical review

This systematic review analyzed previously published literature and did not involve prospective intervention, interaction with human participants, or access to identifiable private information; institutional review board approval and informed consent were therefore not applicable.

## Data availability

The study-selection audit, characteristics of the 18 retained studies, and risk-of-bias appraisal are reported in the appendices and associated review working files. The original study-level screening log for 14 historical full-text exclusions was not available during manuscript preparation.

## Declaration of artificial intelligence (AI) and AI-assisted technologies

During preparation of this work, the authors used an AI-assisted tool for editorial, formatting, and manuscript-organization support. The authors reviewed and edited the content as needed and take full responsibility for the content of the publication.

## Funding

This research did not receive any specific grant from funding agencies in the public, commercial, or not-for-profit sectors.

## Declaration of competing interest

The authors declare that they have no known competing financial interests or personal relationships that could have appeared to influence the work reported in this paper.
