# Supplementary material for: Obesity and cellulitis: Associations with infection severity, recurrence, and healthcare utilization—A systematic review
Source: Obes Pillars. 2026 Oct 13;20:100345. doi: 10.1016/j.obpill.2026.100345 (PMC13697273; doi:10.1016/j.obpill.2026.100345)
Supplement: Supplementary file 2 [file mmc2.docx]

# Appendix 2. Refined Database Search Strategies

| **Database** | **Search terms** |
| --- | --- |
| PubMed | ("Cellulitis"[Mesh] OR "cellulitis" OR "soft tissue infection*" OR "erysipelas" OR "skin infection*") AND ("Obesity"[Mesh] OR "obesity" OR "obese" OR "body mass index" OR "BMI" OR "overweight" OR "adiposity" OR "visceral fat") AND ("hospitalization" OR "hospital length of stay" OR "severity" OR "clinical outcome*" OR "treatment failure" OR "infection severity") |
| Embase | ('cellulitis' OR 'soft tissue infection' OR 'erysipelas' OR 'skin infection') AND ('obesity' OR 'body mass index' OR 'bmi' OR 'obese' OR 'overweight' OR 'adiposity') AND ('hospitalization' OR 'length of stay' OR 'severity' OR 'clinical outcomes' OR 'treatment failure') |
| CINAHL | (('cellulitis' OR 'soft tissue infection' OR 'erysipelas' OR 'skin infection')) AND (('obesity' OR 'body mass index' OR 'bmi' OR 'obese' OR 'overweight' OR 'adiposity')) AND (('hospitalization' OR 'length of stay' OR 'severity' OR 'clinical outcomes' OR 'treatment failure')) |
| Web of Science | TS=(cellulitis OR "skin infection*" OR "soft tissue infection*" OR erysipelas) AND TS=(obesity OR obese OR "body mass index" OR BMI OR overweight OR adiposity) AND TS=("hospitalization" OR "length of stay" OR "severity" OR "clinical outcome*" OR "treatment failure") |
