# Supplementary material for: Obesity and cellulitis: Associations with infection severity, recurrence, and healthcare utilization—A systematic review
Source: Obes Pillars. 2026 Oct 13;20:100345. doi: 10.1016/j.obpill.2026.100345 (PMC13697273; doi:10.1016/j.obpill.2026.100345)
Supplement: Supplementary file 3 [file mmc3.docx]

# Appendix 3. Characteristics of the 18 studies retained in the final evidence synthesis

| **Study** | **Design** | **Journal** | **Key obesity/BMI finding** | **Article** |
| --- | --- | --- | --- | --- |
| 1 — Kozłowska, Dorota (2016) | Retrospective cohort | Przegl Epidemiol | Higher BMI was associated with prolonged hospitalization in patients with erysipelas. | Clinical and epidemiological assessment of patients hospitalized for primary and recurrent erysipelas |
| 5 — Matych, Marta (2025) | Retrospective cohort | Journal of clinical medicine | Obesity was independently associated with prolonged hospitalization for erysipelas. | Primary and Recurrent Erysipelas—Epidemiological Patterns in a Single-Centre Retrospective Analysis |
| 10 — Wiederhold, A. (2024) | Prospective cross-sectional | Antibiotics (Basel) | Obesity was associated with cellulitis diagnosis in a prospective emergency-department cohort. | Characteristics and Antibiotic Treatment of Patients with Cellulitis in the Emergency Department |
| 19 — Chamli, A (2021) | Retrospective cohort | Tunisie Medicale | Obesity/BMI was evaluated among recurrence and severity factors; BMI was not a significant recurrence predictor. | Factors associated with acute and recurrent erysipelas in a young population: a retrospective study of 147 cases |
| 24 — Sapula, M (2020) | Cross-sectional | Interdisciplinary Perspectives on Infectious Diseases | Higher BMI was associated with recurrent erysipelas/cellulitis. | In Search of Risk Factors for Recurrent Erysipelas and Cellulitis of the Lower Limb: A Cross-Sectional Study of Epidemiological Characteristics of Patients Hospitalized due to Skin and Soft-Tissue Infections |
| 26 — Cheong, H.S (2019) | Prospective cohort | Journal of Clinical Medicine | Increasing BMI was associated with incident cellulitis and cellulitis-related hospitalization. | Metabolic obesity phenotypes and risk of cellulitis: A cohort study |
| 34 — Theofiles, M (2015) | Retrospective cohort | Journal of Primary Care & Community Health | BMI ≥50 kg/m² and weight ≥120 kg were associated with greater outpatient cellulitis treatment failure. | Cellulitis in Obesity: Adverse Outcomes Affected by Increases in Body Mass Index |
| 36 — Nassaji, M (2016) | Case-control | Eastern Journal of Medicine | Higher BMI was independently associated with acute cellulitis in case-control analysis. | Risk factors of acute cellulitis in adult patients: A case-control study |
| 47 — Bruun, T (2016) | Prospective cohort | Clinical Infectious Diseases | Higher BMI was associated with poorer early treatment response. | Early Response in Cellulitis: A Prospective Study of Dynamics and Predictors |
| 63 — Harpsøe, M.C (2016) | Prospective cohort | American Journal of Epidemiology | Higher BMI was associated with increased risk of erysipelas hospitalization. | Body Mass Index and Risk of Infections Among Women in the Danish National Birth Cohort |
| 68 — Abetz, J.W (2017) | Retrospective cohort | Emergency Medicine Australia | Obesity independently predicted transfer of care or overstay in an emergency short-stay unit. | Transfer of care and overstay in the management of cellulitis in the emergency short stay unit: A retrospective cohort study |
| 70 — Kaufman, K.R (2016) | Retrospective cohort | Journal of Clinical Pharmacy and Therapeutics | Morbid obesity was not significantly associated with higher cephalexin treatment failure. | Evaluation of cephalexin failure rates in morbidly obese patients with cellulitis |
| 71 — Grinlinton, M (2018) | Retrospective cohort | ANZ Journal of Surgery | Obesity was associated with longer hospital length of stay. | Clinical characteristics affecting length of stay in patients with cellulitis |
| 72 — Carey, I (2025) | Electronic health-record cohort | International Journal of Obesity | Higher BMI was strongly associated with cellulitis incidence in a large electronic-health-record cohort. | Body mass index and infection risks in people with and without type 2 diabetes: A cohort study using electronic health records |
| 73 — Garg, A (2017) | Retrospective cohort | Journal of the American Academy of Dermatology | Obesity was associated with decreased likelihood of discharge/prolonged hospitalization. | Clinical characteristics associated with days to discharge among patients admitted with a primary diagnosis of lower limb cellulitis |
| 74 — Zhang, J (2016) | Retrospective cohort | European Journal of Clinical Microbiology and Infectious Diseases | Obesity was associated with longer outpatient parenteral antimicrobial therapy duration. | OPAT for cellulitis: its benefits and the factors that predispose to longer treatment |
| 75 — Njim, T (2017) | Case-control | BMC Infectious Diseases | Obesity was independently associated with lower-limb cellulitis in case-control analysis. | Risk factors of lower limb cellulitis in a level-two healthcare facility in Cameroon: a case-control study |
| 77 — Garg, A (2017) | Retrospective cohort | Dermatology | Obesity was not significantly associated with lower-limb cellulitis readmission. | Clinical Factors Associated with Readmission among Patients with Lower Limb Cellulitis |

*Risk-of-bias judgments are JBI-informed domain assessments. “Provisional” indicates that a complete primary report was not available for all appraisal domains; unreported domains were treated as unclear rather than favorable. These summaries are not intended to imply a validated numerical JBI quality score.*
