# Supplementary material for: Obesity and cellulitis: Associations with infection severity, recurrence, and healthcare utilization—A systematic review
Source: Obes Pillars. 2026 Oct 13;20:100345. doi: 10.1016/j.obpill.2026.100345 (PMC13697273; doi:10.1016/j.obpill.2026.100345)
Supplement: Supplementary file 4 [file mmc4.docx]

# Appendix 4. Risk-of-bias appraisal of retained studies

| **Study** | **Design** | **Appraisal status** | **Preliminary judgment** | **Appraisal note** |
| --- | --- | --- | --- | --- |
| Kozłowska 2016 | Cohort | Provisional — abstract + journal record | Moderate—provisional | Hospitalized erysipelas cohort and BMI/LOS association are clear; full article should be checked for confounder strategy and completeness before final scoring. |
| Matych 2025 | Cohort | Appraised | Moderate | Clear hospitalized cohort and outcomes; retrospective single-center design leaves residual confounding concerns. |
| Wiederhold 2024 | Cross-sectional | Appraised | Moderate | Prospective multicenter enrollment and expert diagnostic adjudication are strengths; obesity analysis is observational/cross-sectional. |
| Chamli 2021 | Cohort | Appraised | Moderate | Clear hospitalized erysipelas cohort and BMI definition; retrospective design and limited confounder handling for obesity-outcome relationships. |
| Sapuła 2020 | Cross-sectional | Appraised | Moderate | Clear hospitalized population and measured BMI; recurrence comparison is cross-sectional and residual confounding is plausible. |
| Cheong 2019 | Cohort | Appraised | Low | Large prospective cohort, objective BMI, linked outcome ascertainment, and multivariable adjustment. |
| Theofiles 2015 | Cohort | Appraised | Moderate | 637 adult outpatients; objective BMI strata and prespecified failure outcome; multivariable adjustment for age, sex, race, diabetes and tobacco. Retrospective design remains the main limitation. |
| Nassaji 2016 | Case-control | Appraised | Moderate | Cases/controls were age- and sex-matched and logistic regression adjusted key factors; hospital-based selection may limit generalizability. |
| Bruun 2016 | Cohort | Appraised | Low–Moderate | Prospective hospitalized cohort with standardized response assessment; observational design and some residual confounding remain. |
| Harpsøe 2016 | Cohort | Pending full text | Low–Moderate—provisional | Large prospective cohort with registry linkage and Cox models; final JBI item-level review needs full methods/results. |
| Abetz 2017 | Cohort | Provisional — detailed abstract | Moderate—provisional | 451 adults admitted to an emergency short-stay unit; clear overstay/transfer outcome and adjusted analysis. Full text still needed for complete follow-up and missing-data appraisal. |
| Kaufman 2016 | Cohort | Appraised | Moderate | Clear exposure groups and outcome; major limitation is small, underpowered sample and retrospective design. |
| Grinlinton 2019 | Cohort | Provisional — abstract | Moderate—provisional | Adults admitted with cellulitis and LOS outcome are clearly defined; available abstract does not provide enough detail on confounder selection, missing data, or follow-up. |
| Carey 2025 | Cohort | Appraised | Low | Very large EHR cohort, measured BMI, linked primary/hospital outcomes, extensive adjustment, infection-specific analyses. |
| Zhang OPAT 2016 | Cohort | Pending — insufficient methodological detail | Pending | Retrospective OPAT cellulitis study is identifiable and obesity was associated with longer treatment, but accessible material is insufficient for a defensible JBI item-level assessment. |
| Garg LOS 2017 | Cohort | Appraised | Moderate | Large multicenter inpatient cohort with multivariable Cox modeling; retrospective coded-data design and residual confounding are limitations. |
| Njim 2017 | Case-control | Appraised | Moderate | Clear case definition, hospitalized controls, age/sex matching, and multivariable adjustment; single-center hospital-based selection remains a concern. |
| Garg Readmission 2017 | Cohort | Provisional — detailed abstract | Moderate—provisional | Clear lower-limb cellulitis readmission cohort with univariate and multivariable models; full text is still needed for missing-data/follow-up details. |
